# Tail Wags the Dog? Functional Gene Classes Driving Genome-Wide GC Content in *Plasmodium* spp.

**DOI:** 10.1093/gbe/evz015

**Published:** 2019-01-23

**Authors:** Andreina I Castillo, Andrew D L Nelson, Eric Lyons

**Affiliations:** 1 School of Environmental Science, Policy, and Management, University of California, Berkeley; 2 School of Plant Sciences, University of Arizona; 3 BIO5 Institute, School of Plant Sciences, University of Arizona

**Keywords:** GC content, GC bias, Plasmodium, CoGe, comparative genomics, gene function, genome evolution

## Abstract

*Plasmodium* parasites are valuable models to understand how nucleotide composition affects mutation, diversification, and adaptation. No other observed eukaryotes have undergone such large changes in genomic Guanine–Cytosine (GC) content as seen in the genus *Plasmodium* (∼30% within 35–40 Myr). Although mutational biases are known to influence GC content in the human-infective *Plasmodium vivax* and *Plasmodium falciparum*; no study has addressed how different gene functional classes contribute to genus-wide compositional changes, or if *Plasmodium* GC content variation is driven by natural selection. Here, we tested the hypothesis that certain gene processes and functions drive variation in global GC content between *Plasmodium* species. We performed a large-scale comparative genomic analysis using the genomes and predicted genes of 17 *Plasmodium* species encompassing a wide genomic GC content range. Genic GC content was sorted and divided into ten equally sized quantiles that were then assessed for functional enrichment classes. In agreement that selection on gene classes may drive genomic GC content, trans-membrane proteins were enriched within extreme GC content quantiles (Q1 and Q10). Specifically, variant surface antigens, which primarily interact with vertebrate immune systems, showed skewed GC content distributions compared with other trans-membrane proteins. Although a definitive causation linking GC content, expression, and positive selection within variant surface antigens from *Plasmodium vivax*, *Plasmodium berghei*, and *Plasmodium falciparum* could not be established, we found that regardless of genomic nucleotide composition, genic GC content and expression were positively correlated during trophozoite stages. Overall, these data suggest that, alongside mutational biases, functional protein classes drive *Plasmodium* GC content change.

## Introduction

The genus *Plasmodium* is formed of vector-borne obligate parasites capable of infecting a wide range of vertebrates. Notably, five species—*Plasmodium vivax*, *Plasmodium knowlesi*, *Plasmodium malariae*, *Plasmodium ovale*, and *Plasmodium falciparum*—cause disease in humans (malaria). Despite control and eradication efforts, malaria continues to have an enormous impact on global health. In 2015 alone, the World Health Organization reported an estimated 1 million new malaria cases and over 400,000 malaria-related deaths (WHO 2016). Furthermore, *Plasmodium* parasites negatively affect endemic biodiversity. Specifically, the introduction of *Plasmodium relictum* to the Hawaiian islands has caused significant declines in native bird population size (Atkinson et al. 2010; Samuel et al. 2015, 2017). In conjunction with their significance as human and animal pathogens, *Plasmodium* parasites are also valuable models for studying fundamental aspects of genome sequence evolution.

Perhaps one of the most noteworthy features of *Plasmodium* genomes is their rapid change in genomic Guanine–Cytosine (GC) content. Within the span of ∼35–40 Myr (Pacheco et al. 2011), GC content has increased by roughly 30% across the genus. For example, ancestral species such as *Plasmodium gaboni* have a genomic GC content of 17.85%, whereas more recently divergent species such as *P. vivax* have a genomic GC content of 44.87% (Carlton et al. 2008; Sundararaman et al. 2016). Interestingly, coding sequence has a higher GC content than noncoding sequence in all *Plasmodium* genomes thus far sequenced (Castillo et al. 2018).

It has been suggested that mutational biases are driving this genome-wide nucleotide composition change. In a study using mutation accumulation laboratory lines, researchers proposed that an overrepresentation of spontaneous A/T substitutions has pushed the *P. falciparum* genome sequence toward higher AT content (Hamilton et al. 2016). Species closely related to *P. falciparum* (*Laverania* subgenus) are equally AT rich, implying that the substitution bias predates the divergence of the *Laverania* group (Hamilton et al. 2016). It has been hypothesized that a later shift in this mutational bias occurred sometime during *Plasmodium* evolution. Explicitly, *P. vivax* is thought to be subject to increased GC-biased heteroduplex repair and decreased AT-biased mutation (Nikbakht et al. 2015); however, the exact evolutionary mechanisms driving this shift are unknown. Although mutational biases have a clear role in shifting *Plasmodium* GC content, several unanswered questions remain. The evolutionary origin, causes, and mechanisms behind these mutational biases remain poorly understood. Furthermore, we lack insight in how the extreme changes in nucleotide composition influence transcription, translation, and protein structure across orthologs, and how different gene functions might favor or be resilient to nucleotide changes.

In other biological systems, natural selection is thought to have partly driven changes in GC content within certain protein families. For instance, in bacterial genomes, the core genome (genes present in all evaluated strains) is significantly more GC rich than the accessory genome (genes absent in certain strains), largely due to purifying selection constraining GC content on core genes (Bohlin et al. 2017). In humans, functional classes such as “Transcription,” “Signaling and transduction,” “Carbohydrate transport and metabolism,” “Amino acid transport and metabolism,” and “Inorganic ion transport and metabolism” have higher average GC content at the third nucleotide position (GC_3_) than the rest of the genome (D’Onofrio et al. 2007). Other studies have also suggested that lineage-specific differences in genomic GC content might be adaptive to environmental changes (Luo et al. 2015). For example, in plant monocots, it is believed that GC content may have shifted in response to desiccation stress in cold and dry climates (Šmarda et al. 2014). These studies clearly show that in certain lineages and gene functional classes, changes in GC content can be adaptive.

Compared with other eukaryotes, the shift in nucleotide composition seen in the genus *Plasmodium* is unparalleled. Previous evidence shows that *Plasmodium* parasites have developed adaptive strategies to successfully infect both members of their complex life cycle: the *Anopheles* vector and the vertebrate hosts (Molina-Cruz and Barillas-Mury 2014; Scully et al. 2017). Moreover, frequent vertebrate host-switches during *Plasmodium* evolution have also created an opportunity for adaptive change to occur (Stoltzfus and McCandlish 2017). Whether such adaptive change could influence the genome-wide compositional change seen in the genus *Plasmodium* remains to be fully explored.

Within the *Plasmodium* genus, certain protein families are strongly affected by natural selection. For example, *Plasmodium* antigen evolution is thought to occur under intense selective pressure imposed by host immunity (Loy et al. 2017). Among the proteins interacting with the vertebrate immune systems, variant surface antigens (VSAs) have a critical role in parasite virulence and immune evasion. VSAs are gene families characterized by multiple exons, accelerated sequence evolution, rapid gene turnover, and expression encompassing the trophozoite through schizont stages of the *Plasmodium* intraerythrocytic developmental cycle (Chen et al. 1998; Bozdech et al. 2008; Frech and Chen 2013; Yam et al. 2016; Martins et al. 2017). Even though they might share a common evolutionary origin (Janssen et al. 2002, 2004), VSA family members are largely species specific and share little sequence similarity between species. VSAs include species-specific families such as the *Plasmodium* Interspersed Repeats (*pir*) family: *P. vivax* (*vir*) and *Plasmodium berghei* (*bir*); the *P. falciparum*-specific Repetitive Interspersed Family (*rif*) coding for the *rifin* protein; the Subtelomeric Variant Open Reading frame (*stevor*) family; and *var* genes encoding the Erythrocyte Membrane Protein 1 (*PfEMP1*) family. Thus, the VSA proteins may be ideal candidates for driving genome sequence evolution in the genus *Plasmodium*.

Here, we evaluate the hypothesis that the rapid shift in genomic GC content seen in *Plasmodium* parasites is driven, in part, by adaptive changes in certain functional classes of genes. We characterized the contribution of genes belonging to different functional groups to the overall variation of coding GC content seen across *Plasmodium* lineages. Among all assessed groups, we found that trans-membrane proteins, particularly VSAs, had extreme GC content values compared with other functional classes within a species. Trans-membrane proteins represent ∼20–30% of protein coding genes in most *Plasmodium* species (e.g., *P. vivax* [18.89%], *P. berghei* [38.60%], and *P. falciparum* [22.78%]) and contribute ∼10–15% of the bases in the *Plasmodium* genomes (e.g., *P. vivax* [12.24%], *P. berghei* [10.16%], and *P. falciparum* [14.57%]). Consequently, we examined the putative relation between VSA expression during the trophozoite stage of the *Plasmodium* life cycle, signatures of selection, and genic GC content variations.

## Materials and Methods

### Mining *Plasmodium*’s Genomic, Coding, GC_3_, and Genic GC Content Values with CoGe

Representative genomes from the four major *Plasmodium* clades (simian, rodents, *Laverania* subgenus, and birds/reptiles) were obtained from NCBI/GenBank (Clark et al. 2016), PlasmoDB (Aurrecoechea et al. 2009), and GeneDB (Logan-Klumpler et al. 2012). All genomes and annotations were imported to the CoGe platform (Tang and Lyons 2012; Haug-Baltzell et al. 2017; Nelson et al. 2018) (https://genomevolution.org/coge/; last accessed February 2, 2019) and made publicly available. A summary of all imported *Plasmodium* genomes, their CoGe genome IDs, internal CoGe links, and associated publication or bioprojects are provided (supplementary table S1, Supplementary Material online).

**Fig. 1.**
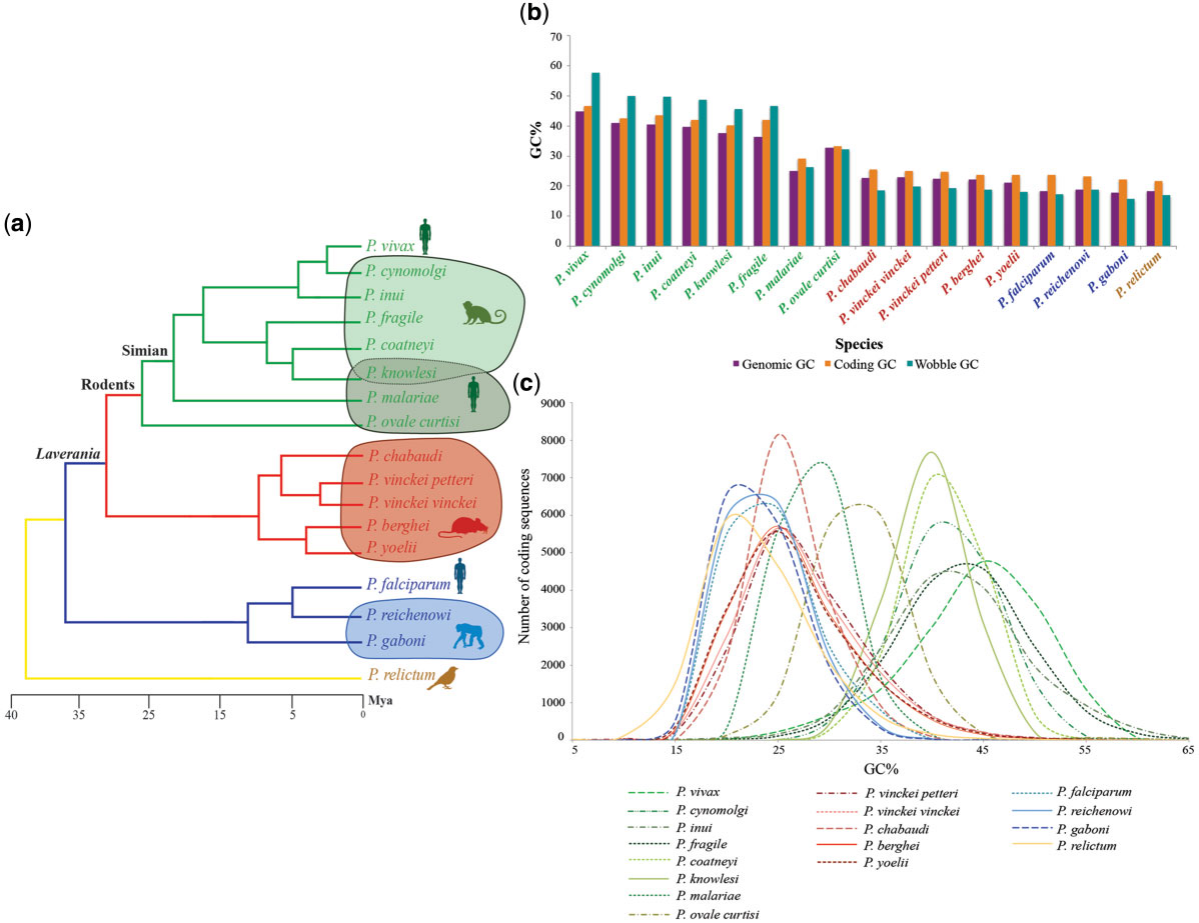
—Characterization of GC content in 17 *Plasmodium* genomes. (*a*) *Plasmodium* phylogeny: simian clade (green), rodent clade (red), *Laverania* subgenus (blue), and *Plasmodium relictum* from the birds/reptiles clade (yellow). (*b*) Histogram of genomic, coding, and GC_3_ content across 17 *Plasmodium* species: genomic GC (purple), coding GC (orange), and GC_3_ (teal). (*c*) GC content distribution of *Plasmodium* CDS. Simian clade: green, rodent clade: red, *Laverania*: blue, and *P. relictum:* yellow.

*Plasmodium* species were organized (fig. 1*a*) according to their phylogenetic relations (Pacheco et al. 2011). CoGe’s GenomeList tool was used to calculate the average genomic, coding, and GC_3_ content across *Plasmodium* species (fig. 1*b*). The CodeOn tool was applied to each genome sequence to characterize the distribution of coding GC content per amino acid. This information was used to estimate the species-specific contribution of individual coding sequences (CDS) to variations in GC content. The number of CDS in 5% GC content increments was calculated for each *Plasmodium* species and normalized by the species with the highest number of reported CDS: *Plasmodium fragile* (fig. 1*c*). The kurtosis, skewedness, deviation from normality (Shapiro–Wilk’s normality test), mean, and standard deviation were estimated for each species using RStudio v3.3 (supplementary table S2, Supplementary Material online). Additionally, the genic GC content for the entire gene repertoire was programmatically accessed from CoGe’s database through CoGe’s REST API (version 1.0) and using custom Python and shell scripts (https://tinyurl.com/y996ygrf; last accessed February 2, 2019).

### GC Content Distribution for Different Functional Classes

**Fig. 2.**
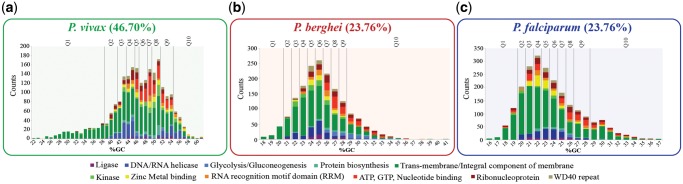
—Histograms of GO-term enrichment within GC content quantiles. Histogram of genes (*y* axis) for bins of GC content (*x* axis). Vertical lines in each histogram delineate GC content quantiles. The number of genes associated with 11 GO-terms is shown as stacked colored bars. (*a*) *Plasmodium vivax* from the simian clade. (*b*) *Plasmodium berghei* from the rodent clade. (*c*) *Plasmodium falciparum* from the *Laverania* subgenus.

Genes were sorted by their GC content and divided into ten equally sized quantiles (∼500–600 genes) within each *Plasmodium* species (fig. 2). The first quantile (Q1) was composed of genes with lowest GC contents and the last quantile (Q10) was composed of genes with highest GC contents. Intermediate quantiles (Q2–Q9) were composed of genes with incrementally higher GC content values. We identified functional annotation clusters that were overrepresented (enriched) within a given GC quantile using the Functional Classification Tool included in the Database for Annotation, Visualization, and Integrated Discovery (DAVID v6.8). DAVID (Huang et al. 2009) was used to identify and group genes with similar annotated functionality. Functional enrichment analyses were performed on *Plasmodium* genomes with annotations and ENTREZ IDs available on NCBI (supplementary tables S3–S5, Supplementary Material online). The ENTREZ IDs were found for all genes within each quantile (Q1–Q10) and used as an input for DAVID’s functional clustering. A variable number of annotation clusters were generated based on the grouped functional categories identified on each quantile. Clusters were organized from those most overrepresented or with higher enrichment scores (ESs) (Annotation Cluster 1) to those least overrepresented or with lowest ESs.

**Fig. 3.**
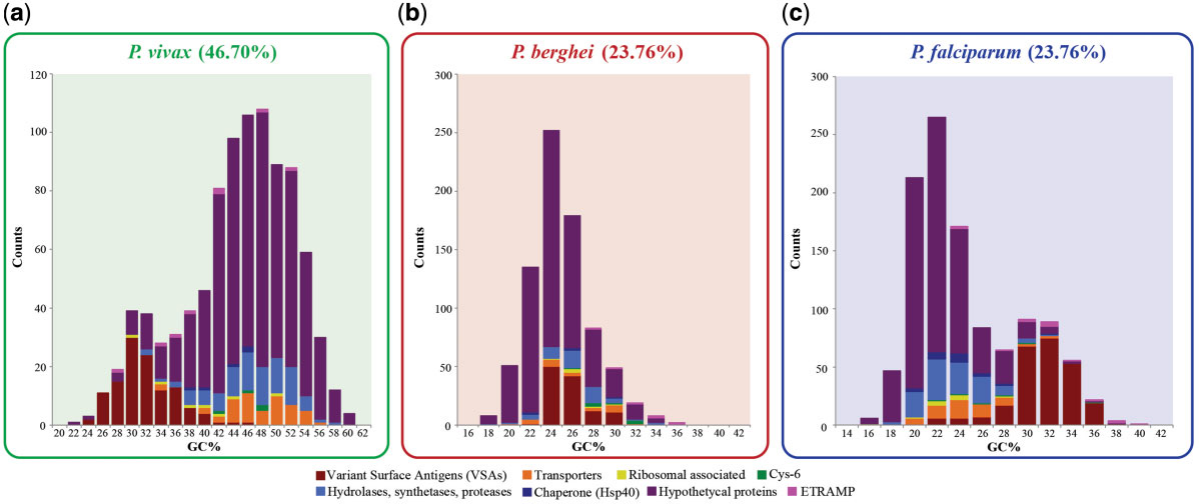
—Histograms of GC content distribution for the top eight trans-membrane protein coding gene functional groups. Trans-membrane functional groups are shown as stacked colored bars in each histogram. Species representing three major *Plasmodium* clades are shown. (*a*) *Plasmodium vivax* from the simian clade. (*b*) *Plasmodium berghei* from the rodent clade. (*c*) *Plasmodium falciparum* from the *Laverania* subgenus.

Clusters defined by the “Trans-membrane/Integral component of membrane” terms tended to have higher ESs in quantiles Q1 and Q10 and thus were analyzed in further detail (fig. 3). The identifying functions of the genes belonging to these annotation clusters were obtained from PlasmoDB using their ENTREZ IDs as queries. Genes belonging to the same protein families or performing similar functions were grouped in sets (supplementary table S6, Supplementary Material online), and their GC content distribution was tabulated. Within the genes identified inside the “Trans-membrane/Integral component of membrane” cluster, members of the VSA multigene families showed the most extreme GC content values. For each species, the differences in GC content distribution between VSA families and an equally sized randomized sample taken from the rest of the coding genome were evaluated with a Wilcoxon rank sum test in RStudio v3.3 (supplementary fig. S7, Supplementary Material online).

### GC Content Distribution and Natural Selection on VSA Families

We assessed if there were signs of positive selective pressure that could hypothetically influence GC content within these families. Species-specific multiple sequence alignments were performed on the following VSA families: *vir* (*P. vivax* Salvador-1 strain [Carlton et al. 2008]); *bir* (*P. berghei* ANKA strain [Khasawneh and Kornreich 2005]); and *stevor*, *rifin*, and *var* (*P. falciparum* 3D7 strain [Gardner et al. 2002]).

**Fig. 4.**
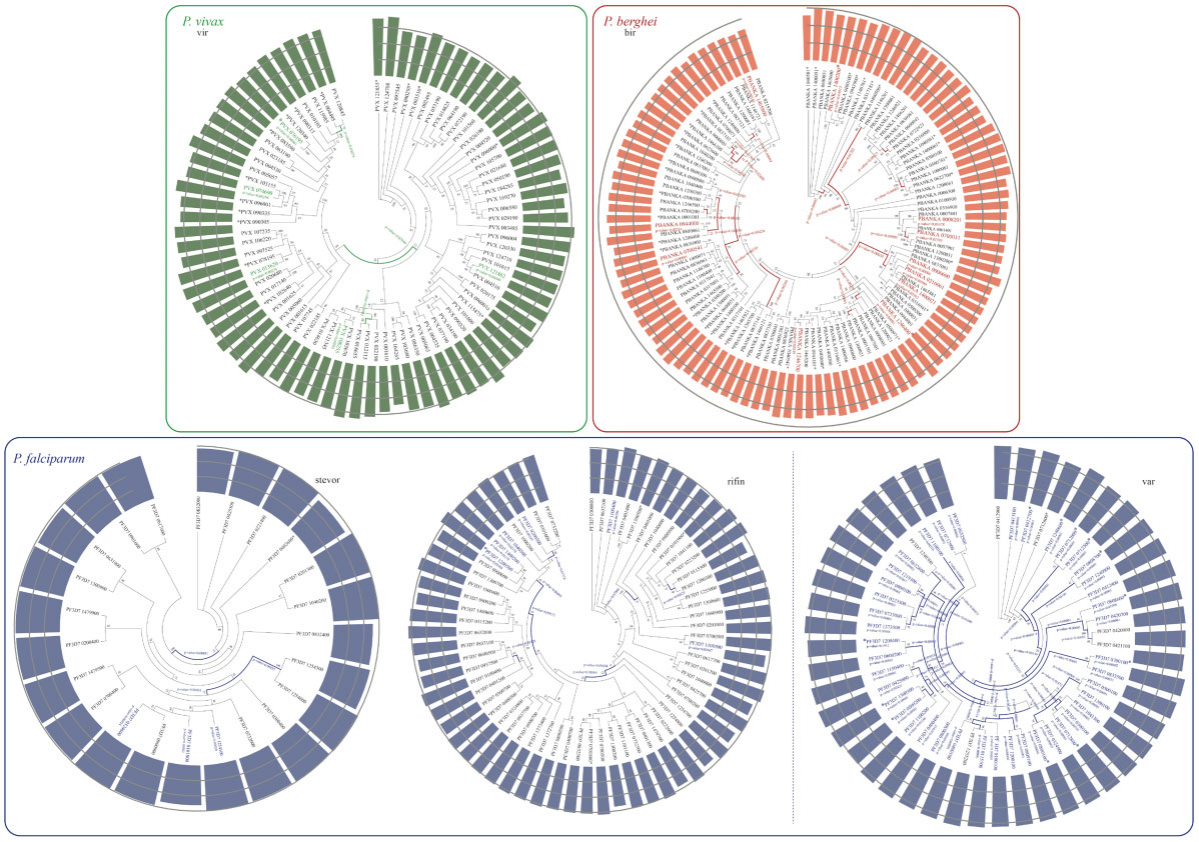
—Relationship between GC content and signatures of selection within the VSA gene families. (*a*) ML tree for *Plasmodium vivax*’s vir family members (green). (*b*) ML tree for *Plasmodium berghei*’s bir family members (red). (*c*) ML tree for *Plasmodium falciparum*’s VSA family members (blue). Bars at the terminal nodes represent genic GC content, with gray circles showing the percent of GC content (10%, 20%, and 30%). Asterisks mark genes expressed during the parasite’s blood cycle. Colored genes and branches indicate significant signs of positive selection.

Codon alignments for the complete CDS of VSA sequences were performed using the MUSCLE algorithm (Edgar 2004) found on MEGA v7 (Kumar et al. 2016). Pseudogenes and incomplete CDS (e.g., sequences missing an exon) were excluded. Maximum likelihood (ML) trees were generated for each VSA family alignment using PhyML (Guindon et al. 2010). Each ML tree used a general time reversible model and 500 bootstrap replicates to evaluate node confidence (fig. 4*a*–*c* and supplementary file S8, Supplementary Material online). A Random Effects Branch-Site model (aBSREL) (Kosakovsky Pond et al. 2011) was employed to detect signatures of positive selection in all branches. The analysis was run using Hyphy’s executable for the command line (Kosakovsky Pond et al. 2005).

### GC Content Distribution and Gene Expression during the Trophozoite Life Stage

**Fig. 5.**
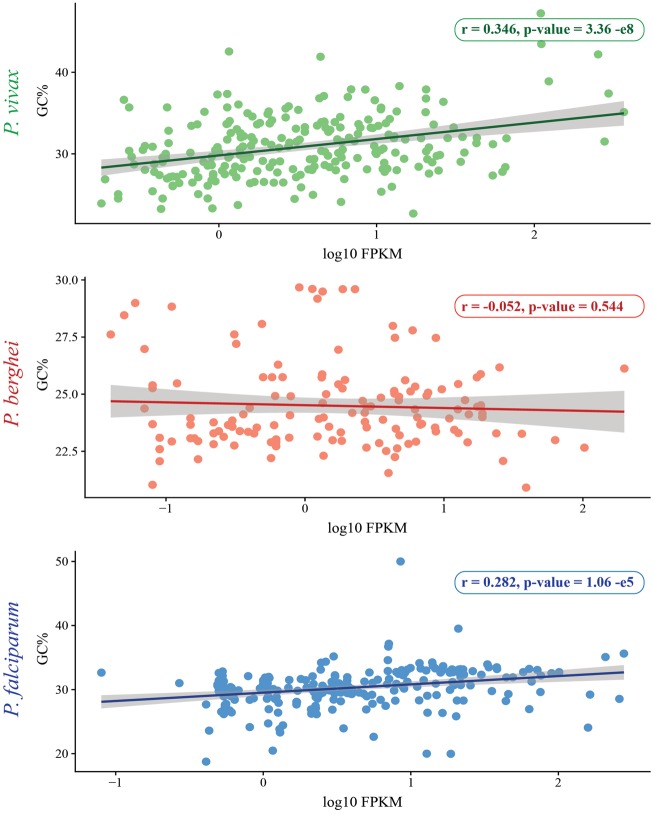
—Relationship between GC content of VSA family members versus FPKM values. Scatterplots and linear regressions with 95% confidence boundaries summarize the distribution of genic GC content and trophozoite log 10 transformed FPKM values in VSA family members.

The relationship between gene expression and GC content was evaluated in the three *Plasmodium* species with publicly available RNA-Seq data: *P. vivax* strain Salvador-1 (Zhu et al. 2016), *P. berghei* strain ANKA (Otto et al. 2014), and *P. falciparum* strain 3D7 (Otto et al. 2010). A significant portion of the *Plasmodium* life cycle is spent consecutively infecting and replicating within vertebrate’s erythrocytes (intraerythrocytic developmental cycle). During this phase, *Plasmodium* parasites are metabolically dependent on the infected erythrocyte and are exposed to host immunity. VSA expression peaks in the *Plasmodium* intraerythrocytic developmental cycle during the trophozoite stage (Chen et al. 1998; Bozdech et al. 2008; Martins et al. 2017). Thus, the Fragments Per Kilobase of transcript per Million mapped reads (FPKM) values for the trophozoite stage were downloaded from PlasmoDB and log 10 transformed. A scatterplot of the genic GC content and log 10 FPKM values was created in RStudio v3.3, and the relation between these two variables was evaluated with a Pearson correlation test. This analysis was conducted both for VSA family members (fig. 5) and for all genes expressed during the trophozoite stage (supplementary fig. S9, Supplementary Material online).

## Results

### Changes in *Plasmodium* Nucleotide Composition Accelerate in the Simian Clade

Coding and GC content at the third nucleotide position (GC_3_) increased by 25% ± 4.26 and 40% ± 6.87, respectively, between ancestral (e.g., *P. relictum*) and recently diverged (e.g., *P. vivax*) species (fig. 1*a* and *b*). With the exception of *P. malariae* (Rutledge et al. 2017), GC content increased between 10 and 14% ± 3.76 in the simian clade compared with other *Plasmodium* clades. These variations were less pronounced (∼1–3% ± 0.44–0.67) within and between the rodent clade, *Laverania* subgenus, and *P. relictum* (fig. 1*b*). Intraclade variations of genomic, coding, and GC_3_ content were noticeable in the simian clade (>10% ± 3.76), even when *P. malariae* was treated as an outlier (>10% ± 2.83) (fig. 1*b*). Moreover, the distribution of GC_3_ content was roughly bimodal in three simian clade species: *P. vivax*, *Plasmodium cynomolgi* (Tachibana et al. 2012), and *Plasmodium coatneyi* (Kissinger et al. 2016) (supplementary fig. S10, Supplementary Material online). Overall, both genome-wide coding and GC_3_ composition are rapidly shifting in the genus, particularly within the simian clade.

### Distinct Functional Classes Uniquely Contribute to Changes in *Plasmodium* GC Content

For all species, the coding GC content displayed a nonstandard distribution that trended toward lower GC values for GC-poor genomes and higher GC values for GC-rich genomes (fig. 1*c* and supplementary table S2, Supplementary Material online). In addition, in some species, the coding GC content distribution was much broader than in others (supplementary table S2, Supplementary Material online). For example, GC-rich species *P. vivax* (46.7 ± 0.19), *P. cynomolgi* (42.63 ± 0.18), and *Plasmodiumfragile* (42.11 ± 0.16) had broad distributions relative to other GC-rich species such as *P. knowlesi* (40.23 ± 0.11) (Pain et al. 2008) and *P. coatneyi* (41.95 ± 0.11) (fig. 1*c*). In sum, coding GC content is rapidly changing in the genus, even between closely related *Plasmodium* species of the simian clade.

The contribution of particular functional classes of genes to the observed shifts in GC content was assessed (fig. 2). The number of genes in *Plasmodium* species ranges between 5,245 and 5,992 (*P. berghei* and *Plasmodium reichenowi*, respectively (Sundararaman et al. 2016)). For each species, the CDS were divided into ten quantiles based on GC content. Quantile 1 (Q1) and Q10 represented the ∼500–600 genes with the most extreme GC content (low or high, respectively) in each genome sequence annotation. The genes within each quantile were analyzed for enrichment in specific functional classes (fig. 2*a*–*c*). Specific GO-terms were enriched in quantiles representing the most extreme GC content values.

In three species of the simian clade (*P. vivax* [fig. 2*a*], *P. cynomolgi* [supplementary fig. S7, Supplementary Material online], and *Plasmodium inui* [supplementary tables S3 and S5, Supplementary Material online]), GO-terms associated with “Trans-membrane/Integral component of membrane” terms were enriched in quantile 1 (Q1). Specifically, the ESs for the “Trans-membrane/Integral component of membrane” term in these species were *P. vivax* (ES = 28.4, *P* value = 7.1 e-32), *P. cynomolgi* (ES = 7.82, *P* value = 1.4 e-09), and *P. inui* (ES = 6.44, *P* value = 5.2 e-09). These genes represent 1.82%, 1.05%, and 1.05% of the total DNA content in the *P. vivax*, *P. cynomolgi*, and *P. inui* genomes, respectively. Q1 represents the set of genes with the lowest GC content for each species. Alternatively, in *P. falciparum* (fig. 2*c*), the closely related term “VSA/Trans-membrane/Integral component of membrane” was enriched in both Q1 (ES = 4.17, *P* value = 8.1 e-07; 2.27% of the genome) and Q10 (ES = 7.59, *P* value = 1.9 e-16; 0.76% of the genome), the latter represents the set of genes with the highest GC content for each species. This result is noteworthy because other enriched GO-terms, that is, “Translation and structural constituents of the ribosome” (*P. berghei* [Q8’s ES =3.83, *P* value = 1.6 e-09; Q9’s ES = 3.89, *P* value = 3.3 e-08; Q10’s ES = 7.6, *P* values = 8.7 e-16]) and “DNA-binding and nucleosome core formation” (*P. berghei* [Q9’s ES = 6.19, *P* value = 4 e-13; Q10’s ES = 4.91, *P* values = 2.9 e-12]; *P. falciparum* [Q10’s ES = 3.5, *P* values = 7 e-05]) did not follow any similar pattern among the examined *Plasmodium* species (supplementary tables S3–S5, Supplementary Material online).

Given their contribution to extreme GC content values in many *Plasmodium* species, genes with an associated “Trans-membrane/Integral component of membrane” term were further clustered based on described gene function (fig. 3). For all evaluated *Plasmodium* species, the largest clusters were composed of trans-membrane proteins with unknown or hypothetical functions (fig. 3*a*–*c*). Among all gene clusters with *identified* functions (supplementary table S6, Supplementary Material online), only the VSA group appears to show skewed GC content distributions compared with the rest of “Trans-membrane/Integral component of membrane” genes (fig. 3*a*–*c*). *Plasmodiumvivax*’s VSA family members (*vir*) had lower GC content values compared with other “Trans-membrane/Integral component of membrane” associated genes (*t* = −29.83, *P* value = 2.2 e-16, fig. 3*a*). In contrast, *P. falciparum* VSA families (*var*, *stevor*, and *rifin*) had higher GC content values in comparison to the rest of “Trans-membrane/Integral component of membrane” associated genes (*t* = 40.31, *P* value = 2.2 e-16, fig. 3*c*). A similar trend was observed for VSA family members in taxa related to *P. vivax* and *P. falciparum*, respectively. Specifically, VSA family members found in *P. cynomolgi* (sister taxa to *P. vivax*) also had lower coding GC values compared with the rest of the genome sequence (supplementary fig. S7b, Supplementary Material online). In contrast, VSA family members found in *Plasmodiumreichenowi* and *Plasmodiumgaboni* (closely related to *P. falciparum*) showed higher coding GC compared with the rest of the genome sequence (supplementary fig. S7*b*, Supplementary Material online). A Wilcoxon rank sum test showed that the compositional differences between VSA family members and the rest of the coding genome were significant for most species (supplementary fig. S7*a*, Supplementary Material online).

### Gene Expression and VSA GC Content, but Not Positive Selection, Are Positively Correlated

Though GC content is shifting in *Plasmodium* parasites, previous studies indicate that a significant portion of the *Plasmodium* genome evolves under strong constraints and purifying selection (Cornejo et al. 2014). Moreover, purifying selection is thought to be intensified by the complex *Plasmodium* parasitic life cycle (Chang et al. 2013). Contrary to the rest of the genome, positive selection has been detected in genes related to the development of antimalarial drug resistance, parasite development in mosquitoes, and variant-expressed multigene families (Park et al. 2012; Cornejo et al. 2014; Duffy et al. 2015). Among these, VSA families are thought to evolve under strong host imposed immune-response selective pressures (Loy et al. 2017); thus, putative signatures of positive selection within VSA family trees were evaluated. In addition, because variations in GC content are known to affect gene expression (Newman et al. 2016), the expression for these gene families was also examined using publicly available RNA-Seq data for the trophozoite stage of *P. vivax*, *P. berghei*, and *P. falciparum* (Otto et al. 2010, 2014; Zhu et al. 2016).

The correlation between VSA genes under positive selection and their expression was assessed (figs. 4 and5). Expressed genes and phylogenetic branches showing significant signs of positive selection were interspersed across both phylogeny and GC content values (fig. 4*a*–*c*). Significant signs of positive selection were found in the *var* family tree (fig. 4*c*) in accordance with previous reports (Plotkin et al. 2004). However, this family also presents the largest number of identified recombination breakpoints compared with other VSAs. It has been suggested that both forces act simultaneously in *var* genes, with mitotic recombination facilitating gene diversity, and positive selection favoring gene variants better equipped to evade host’s immune responses (Bopp et al. 2013; Claessens et al. 2014). Genic GC content was significantly higher for VSA *P. falciparum* genes showing signs of positive selection compared with nonselected genes (*t* = 9.893, *P* value = 6.76 e-16). It was not possible to compare this trend in *P. berghei* or *P. vivax* due to the small number of sequences with signatures of positive selection detected. VSA GC content and expression during the trophozoite stage was positively correlated in both *P. falciparum* (*r* = 0.282, *P* value = 1.06 e-5) and *P. vivax* (*r* = 0.364, *P* value = 3.36 e-8), but not in *P. berghei* (fig. 5). Similarly, a significant and positive correlation between GC content and expression during the trophozoite life cycle stage was observed when examining all genes in all three species (supplementary fig. S9*a*–*c*, Supplementary Material online).

## Discussion

Here, a large-scale comparative genomic approach was used to examine factors influencing observed shifts in genomic GC content within the *Plasmodium* genus (fig. 1 and supplementary table S7, Supplementary Material online). In agreement with previous reports (Castillo et al. 2018), coding GC content in all *Plasmodium* spp. was found to be elevated in comparison to genomic GC content. An interesting pattern where GC_3_ content was higher than genomic and coding GC content in the simian clade, but lower in the rodent clade, *Laverania* subgenus, and the ancestral lineage, *P. relictum*, was also observed. In eukaryotes, protein expression (Yadav and Swati 2012), function (Louie et al. 2003), and structure (Basile et al. 2017) have been known to limit coding GC content, partly due to selective constraints. These selective constraints may result from mutational biases, such as the elevated rate of G/C to A/T transitions compared with the rate of A/T transitions to G/C observed in *P. falciparum* mutation accumulation lines (Hamilton et al. 2016) or the elevated GC_3_ content in *P. vivax* (Nikbakht et al. 2015). The shifts in GC_3_ content observed here suggest that putative differences in mutational bias are not limited to either *P. vivax* or *P. falciparum*, but instead show a strong phylogenetic pattern and likely originate from ancestral events during the evolution of the genus. Furthermore, the bimodal GC_3_ distributions observed in some *Plasmodium* species (supplementary fig. S10, Supplementary Material online) hint to groups of genes with distinct mutational biases or selective pressures within a single species.

Genes with associated GO-terms, “Trans-membrane/Integral component of membrane” was the dominant GO-term recovered for most GC content quantiles (fig. 2). Moreover, this GO-term, which contains genes essential for parasite survival (Sullivan and Krishna 2005; Curtidor et al. 2008; Kirk and Lehane 2014), was highly enriched in boundary GC content quantiles (Q1 and Q10), especially in species with extreme nucleotide compositions. During erythrocyte invasion and intraerythrocytic development (Curtidor et al. 2008), some *Plasmodium* integral membrane proteins are exposed to the immune system, thus providing an opportunity for strong positive selection. In addition, proteins with trans-membrane motifs evolve significantly faster than other *Plasmodium* genes (Carlton et al. 2008). A further examination of this GO-term class revealed that, with some exceptions, GC content was particularly skewed to the extremes in the VSA family of trans-membrane proteins, though a significant number of trans-membrane proteins are annotated as “unknown/hypothetical proteins” (fig. 3). Similar to our observation in species of the subgenus *Laverania*, in *Haemoproteus tartakovskyi* (a bird-infective obligate parasite closely related to the genus *Plasmodium*), VSA family members are enriched for higher GC content relative to other coding genes (Bensch et al. 2016). In contrast, VSA family members of the simian clade harbored a lower GC content relative to the rest of the genome sequence (fig. 3). Thus, regardless of the species, it is clear that VSA genes display GC contents that seem to be at odds with the rest of the genome sequence. This suggests that host-related selective pressures drive rapid sequence evolution, which may drive genomic GC content change in those genes. Nonetheless, it remains unclear if the selection acting on VSA is driving species-level shifts in genomic GC content, or if GC content on these genes is simply lagging behind the rest of the genome sequence.

Attempts to address this question were confounded by the lack of a clear relationship between VSA GC content and signatures of selection at the genic level (fig. 4*a*–*c*). Significant signs of positive selection were interspersed in both internal and terminal branches of each phylogeny, regardless of genic GC content at the tips. This suggests that the enrichment of VSA genes in extreme GC quantiles is not a result of selection along specific phylogenetic branches of gene paralogs. The evidence of selection revealed is likely linked to specific gene functions instead and may hint at rapid diversifying selection operating on many genes simultaneously. An alternative explanation for why VSA GC content appears to be evolving in the opposite direction of other genes may be found in nonadaptive forces such as recombination, GC-biased gene conversion, and local nucleotide biases. Both mitotic recombination (Bopp et al. 2013; Claessens et al. 2014) and variation in local nucleotide composition (Carlton et al. 2008; Tachibana et al. 2012) have been considered as potential factors mediating VSA evolution and might result in the trends observed here.

Interestingly, with the exception of *P. knowlesi* (Pain et al. 2008), *P. coatneyi* (Kissinger et al. 2016), and species from the subgenus *Laverania*, VSA families are located in subtelomeric chromosome regions (Gardner et al. 2002; Carlton et al. 2008; Tachibana et al. 2012; Rutledge et al. 2017). Nucleotide composition in these regions is strongly AT biased in *P. vivax* and *P. cynomolgi* (Carlton et al. 2008; Tachibana et al. 2012), but not so in other *Plasmodium* species (supplementary fig. S11, Supplementary Material online). Significant changes in VSA nucleotide composition compared with the rest of the coding genome were observed in *Plasmodium* species with VSAs clustered in the subtelomeric chromosome regions as well as in species with VSAs distributed across the chromosome length (e.g., *P. falciparum* and *P. knowlesi*). In species with significantly GC-poor subtelomeres (*P. vivax* and *P. cynomolgi*), VSAs were the most numerous protein group within 30 kb of the chromosome ends. In addition, the GC content distribution within this region emulated that previously observed for the Trans-membrane/Integral component of membrane groups, specifically VSAs (figs. 2a and 3a). On the other hand, in GC-rich *Plasmodium* species with clustered subtelomeric VSAs (e.g., *P. malariae*) and species with scattered VSAs (e.g., *P. knowlesi* and *P. coatneyi*), the GC content distribution of CDS within the same 30 kb region emulated that observed on the entire coding genome (supplementary fig. S12, Supplementary Material online). Nonetheless VSAs in these species had significantly smaller GC content differences compared with the rest of the genome (supplementary fig. S7, Supplementary Material online). Overall, though there are some species-specific variations, the GC content of VSA family members remains at odds with the GC content of the rest of the genome sequence, irrespective of their genomic location.

Whether the lower GC content observed in *P. vivax* and *P. cynomolgi* VSA families originates from unique AT-biased mutation patterns within these regions, or if VSA-specific processes drive subtelomeric GC content to lower values is something that remain inconclusive. Regions of lower amino acid diversity compared with other coding regions have previously been described in *P. falciparum* (Zilversmit et al. 2010). Moreover, they have been commonly described in intragenic regions of VSA families across *Plasmodium* species (Janssen et al. 2004; Joannin et al. 2008). Recent studies have found that regions of low complexity tend to remain unchanged over evolutionary time and that composition preferences in the region are unrelated to compositional biases observed in the rest of the genome (Chaudhry et al. 2018). Furthermore, the authors suggest that natural selection might play a key role in determining the amino acid composition in regions of low complexity (Chaudhry et al. 2018). Therefore, it is possible that the nucleotide composition differences in VSAs compared with the rest of the coding genome might be the product of selective forces acting on low complexity region across *Plasmodium* species. Previous work showed that highly expressed genes favor usage of AT-ending codons in both *P. falciparum* and *P. vivax* (Yadav and Swati 2012). Thus, the positive correlation between VSA expression and their GC content observed in *P. vivax* and *P. falciparum* during the trophozoite stage of the *Plasmodium* life cycle is noteworthy (fig. 5) because it contradicts the general trend observed across all genes. In *P. vivax* and *Plasmodium chabaudi* (Khasawneh and Kornreich 2005), some VSAs mediate cytoadherence (Bernabeu et al. 2012), rosette formation, and cell invasion (Yam et al. 2016) and are trafficked to different cellular compartments. A preference for higher GC content in certain VSA family members may thus reflect differential subcellular erythrocyte localization and their unique functions (Bernabeu et al. 2012). In addition, intrafamily differences in GC content might be connected to a proposed strategy whereby *P. falciparum* alters expression patterns of VSA genes to avoid immunological detection and promote survival (Abdi et al. 2016).

Previous studies have led to contradictory conclusions regarding the genome-wide relation between GC content and gene expression. Although some studies show a negative correlation between coding GC content and gene expression in eukaryotes (Rao et al. 2013), others have described a positive correlation between both factors (Kudla et al. 2006). Despite the drastic differences in GC content observed within the *Plasmodium* genus, all tested species displayed a preference for high GC content during the trophozoite stage, which aligns with the latter model (supplementary fig. S9, Supplementary Material online). Previous studies have proposed that *Plasmodium* transcription is highly optimized and that most genes function at multiple points in the life cycle (Bushell et al. 2017). Therefore, it is possible that *Plasmodium* genic GC content changes could be related to mRNA transcription and processing efficiency. Alternatively, although other studies suggest that translational selection has a limited role in shaping global codon and amino acid usage bias within the genus *Plasmodium*; the correlation between amino acid frequency of highly expressed genes and their respective tRNA is slightly higher in some species (Yadav and Swati, 2012). Therefore, it would be of interest to further evaluate this pattern specifically in the VSA gene family. Given the importance of efficiently expressing VSA genes, their translational efficiency may be driving genome-wide changes in GC content. Indeed, the evidence of biased GC content in the subtelomeric genomic regions harboring VSA genes (e.g., *P. vivax*) versus the rest of the genome may be a footprint of this effect. Although the presented data do not refute that hypothesis, additional population-level genomic and functional surveys as well as long-term evolution studies are needed in *Plasmodium*. Ideally, an artificial selection scheme similar to the long-term adaptive evolution studies in *Escherichiacoli* (Blount et al. 2012) would be performed to definitively determine if the tail wags the dog for genomic GC composition evolution in *Plasmodium* species.

In sum, large-scale comparative genomic analyses are powerful means to understand how genome sequences evolve, particularly regarding genome-wide GC content shifts. Nucleotide compositional changes are crucial in genome evolution, phylogenetic reconstruction, and in evaluating expression and translation dynamics. *Plasmodium* genomes are incredibly valuable models to explore the causes and effects of such nucleotide composition shifts. Moreover, the availability of many sequenced genomes within the genus provides a unique resource to better understand the forces driving rapid shifts in genomic GC content. When attempting to understand what is driving differences in genomic GC content within the *Plasmodium* genus, these data suggest that, alongside mutational biases, functional protein classes drive GC content changes across the genome.

## Supplementary Material

Supplementary data are available at *Genome Biology and Evolution* online.

## Supplementary Material

Supplementary Data

## Footnotes

Data deposition: This project used publicly available data and integrated in the comparative genomics platform, CoGe. No new data were generated and thus we cannot use this statement.

## References

[evz015-B1] Abdi AI, et al 2016 Global selection of *Plasmodium falciparum* virulence antigen expression by host antibodies. Sci Rep. 6:1–9. 26804201 10.1038/srep19882 PMC4726288

[evz015-B2] Atkinson CT, Samuel MD, Atkinson CT, Samuel MD. 2010 Nordic Society Oikos Avian malaria *Plasmodium relictum* in native Hawaiian forest birds: epizootiology and demographic impacts on ’apapane *Himatione Sanguinea*. J Avian Biol. 41:357–366.

[evz015-B3] Aurrecoechea C, et al 2009 PlasmoDB: a functional genomic database for malaria parasites. Nucleic Acids Res. 37:539–543. 10.1093/nar/gkn814 PMC2686598 18957442

[evz015-B4] Basile W, Sachenkova O, Light S, Elofsson A. 2017 High GC content causes orphan proteins to be intrinsically disordered. PLoS Comput Biol. 13:1–19. 10.1371/journal.pcbi.1005375 PMC5389847 28355220

[evz015-B5] Bensch S, et al 2016 The genome of *Haemoproteus tartakovskyi* and its relationship to human malaria parasites. Genome Biol Evol. 8:1361–1373. 27190205 10.1093/gbe/evw081 PMC4898798

[evz015-B6] Bernabeu M, et al 2012 Functional analysis of *Plasmodium vivax* VIR proteins reveals different subcellular localizations and cytoadherence to the ICAM-1 endothelial receptor. Cell Microbiol. 14(3):386–400. 22103402 10.1111/j.1462-5822.2011.01726.x

[evz015-B7] Blount ZD, Barrick JE, Davidson CJ, Lenski RE. 2012 Genomic analysis of a key innovation in an experimental *Escherichia coli* population. Nature 489(7417):513. 22992527 10.1038/nature11514 PMC3461117

[evz015-B8] Bohlin J, Eldholm V, Pettersson JHO, Brynildsrud O, Snipen L. 2017 The nucleotide composition of microbial genomes indicates differential patterns of selection on core and accessory genomes. BMC Genomics 18:151. 28187704 10.1186/s12864-017-3543-7 PMC5303225

[evz015-B9] Bopp SE, et al 2013 Mitotic evolution of *Plasmodium falciparum* shows a stable core genome but recombination in antigen families. PLoS Genet. 9(2):e1003293. 23408914 10.1371/journal.pgen.1003293 PMC3567157

[evz015-B10] Bozdech Z, et al 2008 The transcriptome of *Plasmodium vivax* reveals divergence and diversity of transcriptional regulation in malaria parasites. Proc Natl Acad Sci U S A. 105(42):16290–16295. 18852452 10.1073/pnas.0807404105 PMC2571024

[evz015-B11] Bushell E, et al 2017 Functional profiling of a *Plasmodium* genome reveals an abundance of essential genes. Cell 170(2):260–272.e8. 28708996 10.1016/j.cell.2017.06.030 PMC5509546

[evz015-B12] Carlton JM, et al 2008 Comparative genomics of the neglected human malaria parasite *Plasmodium vivax*. Nature 455(7214):757–763. 18843361 10.1038/nature07327 PMC2651158

[evz015-B13] Castillo AI, Nelson ADL, Haug-Baltzell AK, Lyons E. 2018 A tutorial of diverse genome analysis tools found in the CoGe web-platform using *Plasmodium* spp. as a model. Database 2018:1–16.

[evz015-B14] Chang H-H, et al 2013 Malaria life cycle intensifies both natural selection and random genetic drift. Proc Natl Acad Sci U S A. 110(50):20129–20134. 24259712 10.1073/pnas.1319857110 PMC3864301

[evz015-B15] Chaudhry SR, Lwin N, Phelan D, Escalante AA, Battistuzzi FU. 2018 Comparative analysis of low complexity regions in *Plasmodia*. Sci Rep. 8(1):335. 29321589 10.1038/s41598-017-18695-y PMC5762703

[evz015-B16] Chen Q, et al 1998 Developmental selection of *var* gene expression in *Plasmodium falciparum*. Nature 394(6691):392–395. 9690477 10.1038/28660

[evz015-B17] Claessens A, et al 2014 Generation of antigenic diversity in *Plasmodium falciparum* by structured rearrangement of *Var* genes during mitosis. PLoS Genet. 10(12):e1004812. 25521112 10.1371/journal.pgen.1004812 PMC4270465

[evz015-B18] Clark K, Karsch-Mizrachi I, Lipman DJ, Ostell J, Sayers EW. 2016 GenBank. Nucleic Acids Res. 44(D1):D67–D72. 26590407 10.1093/nar/gkv1276 PMC4702903

[evz015-B19] Cornejo OE, Fisher D, Escalante AA. 2014 Genome-wide patterns of genetic polymorphism and signatures of selection in *plasmodium vivax*. Genome Biol Evol. 7:106–119. 25523904 10.1093/gbe/evu267 PMC4316620

[evz015-B20] Curtidor H, et al 2008 Characterization of *Plasmodium falciparum* integral membrane protein Pf25-IMP and identification of its red blood cell binding sequences inhibiting merozoite invasion in vitro. Protein Sci. 17(9):1494–1504. 18556472 10.1110/ps.036251.108 PMC2525525

[evz015-B21] D’Onofrio G, Ghosh TC, Saccone S. 2007 Different functional classes of genes are characterized by different compositional properties. FEBS Lett. 581:5819–5824. 18037382 10.1016/j.febslet.2007.11.052

[evz015-B22] Duffy CW, et al 2015 Comparison of genomic signatures of selection on *Plasmodium falciparum* between different regions of a country with high malaria endemicity. BMC Genomics. 16:1–11. 26173872 10.1186/s12864-015-1746-3 PMC4502944

[evz015-B23] Edgar RC. 2004 MUSCLE: multiple sequence alignment with high accuracy and high throughput. Nucleic Acids Res. 32(5):1792–1797. 15034147 10.1093/nar/gkh340 PMC390337

[evz015-B24] Frech C, Chen N. 2013 Variant surface antigens of malaria parasites: functional and evolutionary insights from comparative gene family classification and analysis. BMC Genomics. 14(1):427. 23805789 10.1186/1471-2164-14-427 PMC3747859

[evz015-B25] Gardner MJ, et al 2002 Genome sequence of the human malaria parasite *Plasmodium falciparum*. Nature 419(6906):498–511. 12368864 10.1038/nature01097 PMC3836256

[evz015-B26] Guindon S, et al 2010 New algorithms and methods to estimate maximum-likelihood phylogenies: assessing the performance of PhyML 3.0. Syst Biol. 59(3):307–321. 20525638 10.1093/sysbio/syq010

[evz015-B27] Hamilton WL, et al 2016 Extreme mutation bias and high AT content in *Plasmodium falciparum*. Nucleic Acids Res. 45(4):1889–1901. 10.1093/nar/gkw1259 PMC5389722 27994033

[evz015-B28] Haug-Baltzell A, Stephens S, Davey S, Scheidegger C, Lyons E. 2017 SynMap2 and SynMap3D: web-based whole-genome synteny browsers. Bioinformatics 33:2197–2198. 28334338 10.1093/bioinformatics/btx144

[evz015-B29] Huang DW, Sherman BT, Lempicki RA. 2009 Systematic and integrative analysis of large gene lists using DAVID bioinformatics resources. Nat Protoc. 4(1):44–57. 19131956 10.1038/nprot.2008.211

[evz015-B30] Janssen CS, Barrett MP, Turner CM, Phillips RS. 2002 A large gene family for putative variant antigens shared by human and rodent malaria parasites. Proc Biol Sci. 269(1489):431–436. 11886633 10.1098/rspb.2001.1903 PMC1690903

[evz015-B30a] Janssen CS, Phillips RS, Turner CMR, Barrett MP. 2004 *Plasmodium* interspersed repeats: the major multigene superfamily of malaria parasites. Nucleic Acids Res. 32(19):5712–5720. 15507685 10.1093/nar/gkh907 PMC528792

[evz015-B31] Joannin N, Abhiman S, Sonnhammer EL, Wahlgren M. 2008 Sub-grouping and sub-functionalization of the RIFIN multi-copy protein family. BMC Genomics. 9(1):19. 18197962 10.1186/1471-2164-9-19 PMC2257938

[evz015-B32] Khasawneh R, Kornreich R. 2005 A comprehensive survey of the *Plasmodium* life cycle by genomic, transcriptomic, and proteomic analyses. Pharmacogenomics 3:781–791. 10.1126/science.1103717 15637271

[evz015-B33] Kirk K, Lehane AM. 2014 Membrane transport in the malaria parasite and its host erythrocyte. Biochem J. 457(1):1–18. 24325549 10.1042/BJ20131007

[evz015-B34] Kissinger JC, et al 2016 High-quality genome assembly and annotation for *Plasmodium*. Genome Announc. 4:4–5. 10.1128/genomeA.00883-16 PMC5009967 27587810

[evz015-B35] Kosakovsky Pond SL, Frost SDW, Muse SV. 2005 HyPhy: hypothesis testing using phylogenies. Bioinformatics 21(5):676–679. 15509596 10.1093/bioinformatics/bti079

[evz015-B36] Kosakovsky Pond SL, et al 2011 A random effects branch-site model for detecting episodic diversifying selection. Mol Biol Evol. 28(11):3033–3043. 21670087 10.1093/molbev/msr125 PMC3247808

[evz015-B37] Kudla G, Lipinski L, Caffin F, Helwak A, Zylicz M. 2006 High guanine and cytosine content increases mRNA levels in mammalian cells. PLoS Biol. 4:0933–0942. 10.1371/journal.pbio.0040180 PMC1463026 16700628

[evz015-B38] Kumar S, Stecher G, Tamura K. 2016 MEGA7: molecular evolutionary genetics analysis version 7.0 for bigger datasets. Mol Biol Evol. 33(7):1870–1874. 27004904 10.1093/molbev/msw054 PMC8210823

[evz015-B39] Logan-Klumpler FJ, et al 2012 GeneDB—an annotation database for pathogens. Nucleic Acids Res. 40:98–108. 10.1093/nar/gkr1032 PMC3245030 22116062

[evz015-B40] Louie E, Ott J, Majewski J. 2003 Nucleotide frequency variation across human genes. Genome Res. 13(12):2594–2601. 14613976 10.1101/gr.1317703 PMC403801

[evz015-B41] Loy DE, et al 2017 Out of Africa: origins and evolution of the human malaria parasites *Plasmodium falciparum* and *Plasmodium vivax*. Int J Parasitol. 47(2–3):87–97. 27381764 10.1016/j.ijpara.2016.05.008 PMC5205579

[evz015-B42] Luo H, Thompson LR, Stingl U, Hughes AL. 2015 Selection maintains low genomic GC content in marine SAR11 lineages. Mol Biol Evol. 32(10):2738–2748. 26116859 10.1093/molbev/msv149

[evz015-B43] Martins RM, et al 2017 An ApiAP2 member regulates expression of clonally variant genes of the human malaria parasite *Plasmodium falciparum*. Sci Rep. 7:1–10. 29070841 10.1038/s41598-017-12578-y PMC5656681

[evz015-B44] Molina-Cruz A, Barillas-Mury C. 2014 The remarkable journey of adaptation of the *Plasmodium falciparum* malaria parasite to New World anopheline mosquitoes. Mem Inst Oswaldo Cruz 109(5):662–667. 25185006 10.1590/0074-0276130553 PMC4156459

[evz015-B45] Nelson ADL, Haug-Baltzell AK, Davey S, Gregory BD, Lyons E. 2018 EPIC-CoGe: managing and analyzing genomic data. Bioinformatics 34(15):2651–2653. 29474529 10.1093/bioinformatics/bty106 PMC6061785

[evz015-B46] Newman ZR, Young JM, Ingolia NT, Barton GM. 2016 Differences in codon bias and GC content contribute to the balanced expression of TLR7 and TLR9. Proc Natl Acad Sci U S A. 113(10):E1362–E1371. 26903634 10.1073/pnas.1518976113 PMC4791032

[evz015-B47] Nikbakht H, Xia X, Hickey DA. 2015 The evolution of genomic GC content undergoes a rapid reversal within the genus *Plasmodium*. Genome 511:1–5. 10.1139/gen-2014-0158 25633864

[evz015-B48] Otto TD, et al 2010 New insights into the blood-stage transcriptome of *Plasmodium falciparum* using RNA-Seq. Mol Microbiol. 76(1):12–24. 20141604 10.1111/j.1365-2958.2009.07026.x PMC2859250

[evz015-B49] Otto TD, et al 2014 A comprehensive evaluation of rodent malaria parasite genomes and gene expression. BMC Biol. 12:86. 25359557 10.1186/s12915-014-0086-0 PMC4242472

[evz015-B50] Pacheco MA, et al 2011 Timing the origin of human malarias: the lemur puzzle. BMC Evol Biol. 11(1):299. 21992100 10.1186/1471-2148-11-299 PMC3228831

[evz015-B51] Pain A, et al 2008 The genome of the simian and human malaria parasite *Plasmodium knowlesi*. Nature 455(7214):799–803. 18843368 10.1038/nature07306 PMC2656934

[evz015-B52] Park DJ, et al 2012 Sequence-based association and selection scans identify drug resistance loci in the *Plasmodium falciparum* malaria parasite. Proc Natl Acad Sci U S A. 109(32):13052–13057. 22826220 10.1073/pnas.1210585109 PMC3420184

[evz015-B53] Plotkin JB, Dushoff J, Fraser HB. 2004 Detecting selection using a single genome sequence of *M. tuberculosis* and *P. falciparum*. Nature 428(6986):942–945. 15118727 10.1038/nature02458

[evz015-B54] Rao YS, Chai XW, Wang ZF, Nie QH, Zhang XQ. 2013 Impact of GC content on gene expression pattern in chicken. Genet Sel Evol. 45:1–7. 23557030 10.1186/1297-9686-45-9 PMC3641017

[evz015-B55] Rutledge GG, et al 2017 *Plasmodium malariae* and *P. ovale* genomes provide insights into malaria parasite evolution. Nature 542(7639):101–104. 28117441 10.1038/nature21038 PMC5326575

[evz015-B56] Samuel MD, Woodworth BL, Atkinson CT, Hart PJ, LaPointe DA. 2015 Avian malaria in Hawaiian forest birds: infection and population impacts across species and elevations. Ecosphere 6:1–21.

[evz015-B57] Samuel MD, et al 2017 The dynamics, transmission, and population impacts of avian malaria in native Hawaiian birds: a modeling approach. Ecol Appl. 21:2960–2973.

[evz015-B58] Scully EJ, Kanjee U, Duraisingh MT. 2017 Molecular interactions governing host-specificity of blood stage malaria parasites. Curr Opin Microbiol. 40:21–31. 29096194 10.1016/j.mib.2017.10.006 PMC5733638

[evz015-B59] Šmarda P, et al 2014 Ecological and evolutionary significance of genomic GC content diversity in monocots. Proc Natl Acad Sci U S A. 111(39):E4096–E4102. 25225383 10.1073/pnas.1321152111 PMC4191780

[evz015-B60] Stoltzfus A, McCandlish DM. 2017 Mutational biases influence parallel adaptation. Mol Biol Evol. 34(9):2163–2172. 28645195 10.1093/molbev/msx180 PMC5850294

[evz015-B61] Sullivan DJ, Krishna S. 2005 Malaria drugs, disease, and post-genomic biology. Berlin Heidelberg New York: Springer.

[evz015-B62] Sundararaman SA, et al 2016 Genomes of cryptic chimpanzee *Plasmodium* species reveal key evolutionary events leading to human malaria. Nat Commun. 7:11078. 27002652 10.1038/ncomms11078 PMC4804174

[evz015-B63] Tachibana S-I, et al 2012 *Plasmodium cynomolgi* genome sequences provide insight into *Plasmodium vivax* and the monkey malaria clade. Nat Genet. 44(9):1051–1055. 22863735 10.1038/ng.2375 PMC3759362

[evz015-B64] Tang H, Lyons E. 2012 Unleashing the genome of *Brassica Rapa*. Front Plant Sci. 3:1–12. 22866056 10.3389/fpls.2012.00172 PMC3408644

[evz015-B65] WHO. 2016 World malaria report. Genève, Switzerland: Global Malaria Programme World Health Organization.

[evz015-B66] Yadav MK, Swati D. 2012 Comparative genome analysis of six malarial parasites using codon usage bias based tools. Bioinformation 8(24):1230–1239. 23275725 10.6026/97320630081230 PMC3530877

[evz015-B67] Yam XY, et al 2016 Characterization of the *Plasmodium* Interspersed Repeats (PIR) proteins of *Plasmodium chabaudi* indicates functional diversity. Sci Rep. 6:1–13. 26996203 10.1038/srep23449 PMC4800443

[evz015-B68] Zhu L, et al 2016 New insights into the *Plasmodium vivax* transcriptome using RNA-Seq. Sci Rep. 6:20498. 26858037 10.1038/srep20498 PMC4746618

[evz015-B69] Zilversmit MM, et al 2010 Low-complexity regions in *Plasmodium falciparum*: missing links in the evolution of an extreme genome. Mol Biol Evol. 9:2198–2209. 10.1093/molbev/msq108 PMC2922621 20427419

